# The Functional Significance of Cardiac Looping: Comparative Embryology, Anatomy, and Physiology of the Looped Design of Vertebrate Hearts

**DOI:** 10.3390/jcdd11080252

**Published:** 2024-08-17

**Authors:** Jörg Männer

**Affiliations:** Group Cardio-Embryology, Institute of Anatomy and Cell Biology, UMG, Georg-August-University Goettingen, D-37075 Goettingen, Germany; jmaenne@gwdg.de

**Keywords:** embryology, cardiac looping, vertebrate hearts, fish hearts, comparative anatomy, functional morphology, pumping function

## Abstract

The flow path of vertebrate hearts has a looped configuration characterized by curved (sigmoid) and twisted (chiral) components. The looped heart design is phylogenetically conserved among vertebrates and is thought to represent a significant determinant of cardiac pumping function. It evolves during the embryonic period of development by a process called “cardiac looping”. During the past decades, remarkable progress has been made in the uncovering of genetic, molecular, and biophysical factors contributing to cardiac looping. Our present knowledge of the functional consequences of cardiac looping lags behind this impressive progress. This article provides an overview and discussion of the currently available information on looped heart design and its implications for the pumping function. It is emphasized that: (1) looping seems to improve the pumping efficiency of the valveless embryonic heart. (2) bilaterally asymmetric (chiral) looping plays a central role in determining the alignment and separation of the pulmonary and systemic flow paths in the multi-chambered heart of tetrapods. (3) chiral looping is not needed for efficient pumping of the two-chambered hearts of fish. (4) it is the sigmoid curving of the flow path that may improve the pumping efficiency of lower as well as higher vertebrate hearts.

## 1. Introduction

Cardiac looping is a morphogenetic process that occurs during the early stages of the development of vertebrate embryonic hearts when these hearts have the relatively simple design of an actively pulsating tubular blood vessel. Looping morphogenesis leads to the transformation of the initially straight embryonic heart tube into a looped tube whose geometric configuration is described in a simplified, two-dimensional fashion as an ‘S-shaped’ loop since the early 19th century [1,2,3,4,5]. The ‘S-shaped’ configuration of the vertebrate embryonic heart loop principally persists during the whole lifespan of vertebrates since it is the first manifestation of the curved and twisted flow paths found in mature vertebrate hearts. Due to the relatively large body size and easy accessibility of chick embryos, the first historically documented observations of embryonic heart loops were made in these higher vertebrates (for review, see [6]). During the past 200 years, however, looping morphogenesis of the embryonic heart tube has been observed in a wide spectrum of extant vertebrate species. This spectrum not only includes land-living vertebrates but also phylogenetically ‘primitive’ and ‘modern’ fish species [1,4,7,8,9,10,11,12]. Moreover, it has been found that several components of the looping process principally run the same way in all vertebrate species studied thus far [6,13,14,15]. This observation not only provides an explanation for the current usage of zebrafish embryos as models for studying the genetic, cellular, and biophysical control of the early morphogenesis of the vertebrate heart [16,17,18,19]. It also suggests that cardiac looping may fulfill one or more phylogenetically conserved functions.

In the past, the fascination of developmental biologists for cardiac looping arose from the fact that the embryonic heart is the first inner organ of vertebrates that adopts a bilaterally asymmetric configuration. Studying the looping embryonic heart may provide central pieces for the completion of a still incomplete jigsaw puzzle, which, upon completion, may explain the development of visceral asymmetries in vertebrates. Moreover, it was found that disturbed left-right patterning of vertebrate embryos led to the development of severe congenital heart defects [15,20,21]. It is, therefore, no wonder that the current research on cardiac looping mainly focuses on the biophysical, cellular, and genetic factors driving the bilaterally asymmetric morphogenesis of the early embryonic heart [17,22,23]. Compared to these currently very productive studies, only relatively few studies have focused on the functional significance of the looped design of embryonic and mature vertebrate hearts [24,25,26,27,28]. Thus, our current knowledge about the functional consequences of this early morphogenetic process seems to lag behind the recent progress made in uncovering its biological drivers. The intent of the present article, therefore, is not only to provide an overview and discussion of the currently available information about the looped design of vertebrate hearts and its functional implications but also to stimulate future research in this area. As already mentioned, studies on fish have significantly contributed to our current understanding of the biological drivers of cardiac looping. In the present article, it will be shown that studies on the cardiovascular system of fish can also provide new insight into the functional consequences of cardiac looping. The article consists of three sections: (1) an overview of the early morphogenesis of vertebrate hearts, with a special focus on comparative aspects of cardiac looping among vertebrates. (2), an overview of the comparative anatomy of mature vertebrate hearts, which focuses on the morphological features resulting from cardiac looping. (3) an overview and discussion of functional interpretations of the looped design of embryonic and mature vertebrate hearts.

## 2. Overview of the Early Morphogenesis of Vertebrate Hearts

**Figure 1:**
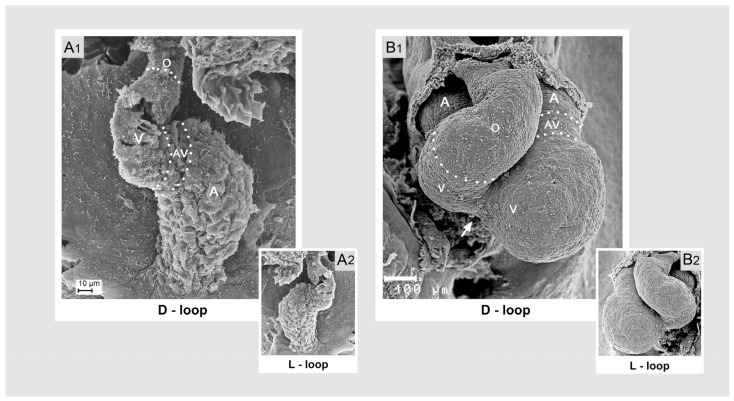
These scanning electron microscopic images depict the normal (D-loop, (**A1**,**B1**)) as well as mirror-imaged configuration (L-loop, (**A2**,**B2**)) of the ‘S-shaped’ heart loop of vertebrates as seen in a zebrafish embryo at 48 h after fertilization (**A1**,**A2**) and in a mouse embryo at 9.5 days after fertilization (**B1**,**B2**). Heart loops are shown in frontal views. The dotted lines mark the borders between the building units of the embryonic heart, which are, in a proximal to distal order, the common atrium (A), the atrioventricular canal (AV), the ventricular bend (V), and the outflow tract (O). The borders between these building units are externally marked by furrows and kinks. Note that in the mouse heart, the common atrium has a right and a left-sided component, which represent the future right and left atrial appendages, respectively. Furthermore, the ventricular bend consists of proximal and distal components, which represent the embryonic left and right ventricles, respectively. The border between the embryonic ventricles is marked by an interventricular furrow (arrow).

In vertebrates, the heart is the first organ to form and function during the embryonic period of development. The human embryonic heart, for example, starts its pumping action during the fourth week after fertilization, which corresponds to the second week after the expected onset of the first missed menstrual bleeding [29]. During this early phase of prenatal cardiogenesis, the shape and pumping action of vertebrate hearts differ markedly from those found in mature hearts. The latter are characterized by the presence of chambers and valves [30], and their mode of generating unidirectional blood flow may be characterized as *valve supported pumping* [27]. The early embryonic heart of vertebrates, in contrast, has the design of a tubular blood vessel that lacks valves. Its mode of pumping action, therefore, has been characterized as *valveless pumping* (for review, see [31]). The valveless heart tube of vertebrate embryos arises from the union of bilaterally paired areas of the lateral plate mesoderm along the ventral midline of the foregut [32]. These areas of the lateral plate mesoderm are called the left and right heart fields. Their union starts with the formation of a straight heart tube, which is aligned along the body midline and becomes connected to the venous and arterial branches of the embryonic cardiovascular system at the caudal and cranial ends, respectively. The future atrial and ventricular heart chambers, therefore, have an original caudal (atria) and cranial (ventricles) identity. Subsequent to its establishment, the straight heart tube elongates by the continuous addition of the new heart field-derived material to its arterial and venous poles. These heart field-derived materials are nowadays named the anterior and posterior second heart fields, while the materials forming the first portion of the embryonic heart tube are named the first heart field [33]. The elongation of the tubular embryonic heart is accompanied by striking changes in its configuration. The initially straight and almost bilaterally symmetric tube now adopts the configuration of a looped tube. The fully looped heart tube is frequently called the ‘S-shaped’ heart loop since its shape is said to resemble the Latin letter S when viewed in a frontal projection. The S-shaped heart loop is a hand-held geometrical object. This means that it has a bilaterally asymmetric configuration, which can principally occur in two chiral variants, named the D-loop and the L-loop enantiomorph (Figure 1).

In the D-loop enantiomorph, the ventricular (cranial) portion of the S-shaped heart loop is bend toward the right side of the body (dextro-loop) (Figure 1(A1,B1)), while the ventricular portion of the L-loop enantiomorph is bend toward the left body side (levo-loop) (Figure 1(A2,B2)). In all vertebrate species studied so far, there is normally a very strong bias toward the development of the D-loop enantiomorph, so the spontaneous occurrence of L-loops is normally a rare event (see Figure 4 in [14]). The D-loop enantiomorph of vertebrate embryonic hearts, therefore, is classified as a fixed or *directional asymmetry*, which strongly suggests that the direction of asymmetric heart looping is inherited [14].

### Comparative Aspects of Cardiac Looping among Vertebrates

As already mentioned, looping morphogenesis of the tubular embryonic heart has been observed in embryos from all classes of extant vertebrates (fishes, amphibia, reptiles, birds, and mammals) studied during the past 200 years. It is, therefore, generally said that cardiac looping is a phylogenetically conserved morphogenetic process among vertebrates. Studies on vertebrate cardiogenesis, however, have also shown that the morphological phenotype of ‘S-shaped’ heart loops can differ considerably between different species [18,23,34] (Figure 1). These differences are based on the fact that cardiac looping is a process that can produce complex three-dimensional configurations that cannot be ascribed simply to form changes along the left-right body axis [23,34,35,36] and cannot be fully defined by the terms D- and L-loops [36].

To make easier the comparison of three-dimensional heart looping between different species, I find it helpful to characterize the complex positional and morphological changes of the looping embryonic heart tube with regard to the main body axes of vertebrates. We, thereby, can distinguish between the four different components of cardiac looping as follows:

(1) positional and morphological changes along the original dorsoventral heart axis.
(2) positional and morphological changes along the craniocaudal body axis.
(3) positional and morphological changes along the so-called left-right body axis, which lead to a bilaterally asymmetric heart shape. This component may be named “lateral looping” or “chiral looping”.
(4) positional and morphological changes that reduce the degree of lateral/chiral looping reached earlier stages of cardiac looping. This component may be named “final positional adjustments”.

**Figure 2:**
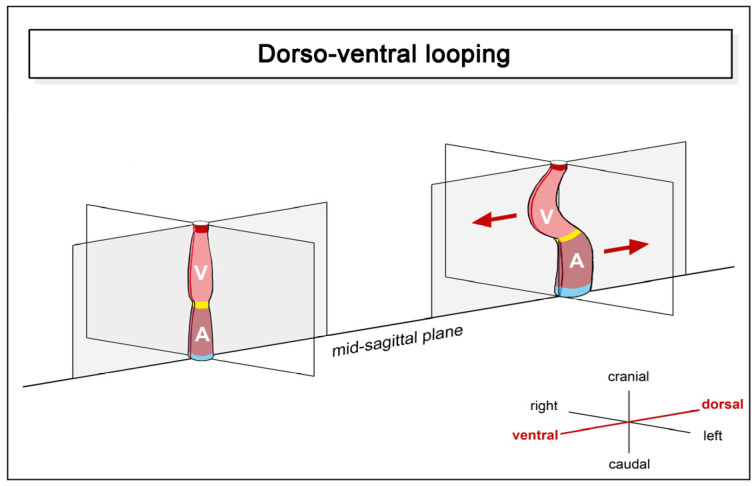
This schematic drawing illustrates the positional and morphological changes of the looping embryonic heart tube along the original ventrodorsal heart axis (indicated by red arrows). These positional changes are mainly driven by ventral bending of the ventricular portion. Color code: blue = confluence of veins; brown = atrial segment; yellow = AV-canal; pink = ventricular segment; red = ventricular outflow element. Abbreviations as used before.

The above-mentioned list of the four looping components should not be understood as showing the normal developmental sequence of cardiac looping. Some of these changes can occur in species-specific manners at the same time [23,34]. Furthermore, simulations have shown that these looping components do not have to arise from different morphogenetic processes. This means that one biophysical mechanism can drive more than one of the four above-mentioned positional and morphological changes in the looping heart [37,38]. Thus, the above-mentioned four looping components may not represent different morphogenetic processes. The purpose of distinguishing four artificially separated ‘events’ is only to make the understanding and comparison of early cardiac morphogenesis among vertebrates easier. In the following, the four components of cardiac looping will be described in detail using a simplified geometrical model that consists, from the beginning, of all building units of the embryonic heart.

(1), **dorsoventral looping**: this component is mainly characterized by a change in the positional relationship of the future atrial and ventricular heart chambers along the original dorsoventral heart axis. Initially, this axis corresponds to the future dorsoventral body axis. However, during lateral/chiral looping (see below), which frequently coincides with dorsoventral looping, the original dorsoventral polarization of the heart tube becomes obscured by rightward rotation. The present scheme of dorsoventral looping (Figure 2), therefore, shows an artificial situation that omits the changes caused by lateral/chiral looping.

**Figure 3:**
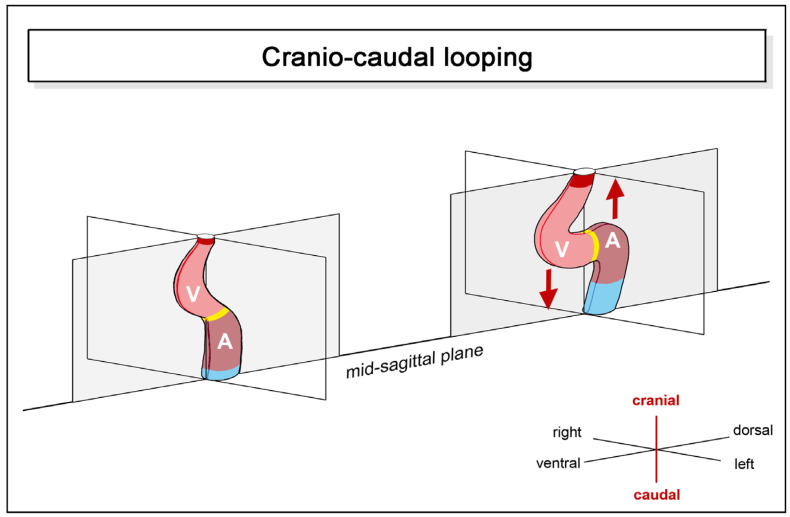
This schematic drawing illustrates the positional and morphological changes of the looping embryonic heart tube along the original craniocaudal body axis. Color codes and abbreviations as used before.

In the pre-looping state, the atrial and ventricular portions of the heart tube are aligned along the craniocaudal axis, with the atrial portion lying caudal to the ventricular portion. The heart tube, therefore, appears as a straight tube. During dorsoventral looping, the ventricular portion underperforms a ventral shift, and the atrial portion undergoes a dorsal shift. These positional changes are driven mainly by ventral bending of the ventricular portion of the heart tube. As a consequence of dorsoventral looping, the atrial portion acquires a dorso-caudal position in relation to the ventricular portion, and the entire heart tube and its main flow path start to adopt the shape of the Latin letter S.

(2), **craniocaudal looping**: the positional and morphological changes characterizing this component may be described as ascent of the atrial portion and/or descent of the ventricular portion of the heart loop along the craniocaudal body axis (Figure 3).

**Figure 4:**
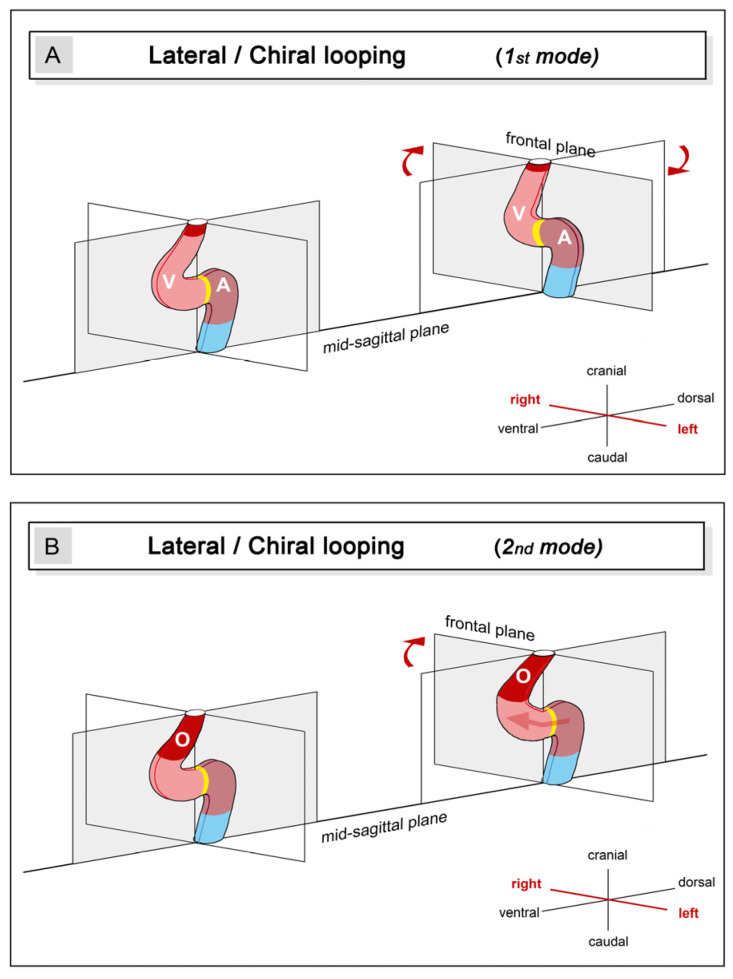
These schematic drawings illustrate the positional and morphological changes of the looping embryonic heart tube along the left-right body axis, so-called lateral or chiral looping. Principally, two different modes of chiral looping can be found in vertebrates (**A**,**B**). The first mode is characterized by rightward rotation of the entire heart around its original longitudinal axis (**A**). Hearts that loop according to this mode have only a very short outflow element. In the second mode, only the ventricular bend and its outflow element undergo rightward rotation (**B**). Hearts that loop in this mode have a substantial myocardial outflow element (O). Color codes and abbreviations as used before.

The atrial ascent/ventricular descent brings the atrial portion of the heart tube into its mature topographical relation to the ventricular portion, which is said to be dorsal to the ventricle in most fishes and dorso-cranial to the ventricle in tetrapods [28,30,39,40]. Atrial ascent/ventricular descent forces the S-shaped deformation of the heart loop and produces the U-shaped ventricular flow path (U-turn loop) usually found in the mature heart of higher vertebrates [28,40]. Observations on sturgeon embryos have shown that the outcome of craniocaudal looping strongly depends on the growth dynamics of the embryonic pericardial cavity. In this species, cardiac looping is interrupted by a developmental phase in which the already acquired configuration of an S-shaped loop practically disappears due to stretching of the heart caused by an increase in the craniocaudal length of the pericardial cavity. The definitive ‘S’-shaped’ loop is then formed during the subsequent phase of forced elongation of the heart [11].

(3), **lateral or chiral looping**: the positional and morphological changes characterizing this component may be described, in a simplified fashion, as resulting from the rightward rotation of the heart loop around its original longitudinal (craniocaudal) axis. Chiral looping differs remarkably between embryonic hearts, in which the ventricular portion is connected to the future arterial root only by a vestigial myocardial outflow element (hearts of jawless fish and modern ray-finned fish), and those in which the ventricular portion connects to the future arterial root via a substantial myocardial outflow element (hearts of cartilaginous fish, primitive ray-finned fish, lobe-finned fish, and tetrapods). In the first group, rightward rotation affects the entire heart tube. As a consequence, its ventricular portion lies to the right of the body midline, and its atrial portion lies to the left of the body midline (Figure 1(A1) and Figure 4A).

**Figure 5:**
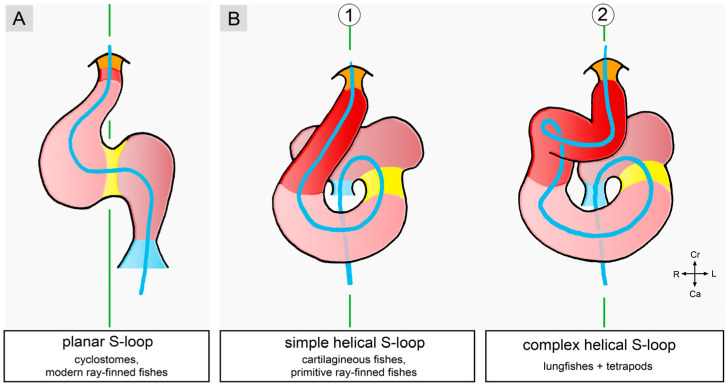
These schematic drawings depict the three basic types of ‘S-shaped’ heart loops found in vertebrates: (**A**) ‘plane’ S-loop, (**B**, No. 1) simple helical S-loop, and (**B**, No. 2) complex helical S-loop. The blue lines depict the cardiac flow paths. The green lines mark the body midline. Based on our own image data from zebrafish (**A**) and data from [11,43] (**B**, No. 1) and [9,41] (**B**, No. 2). Color codes as used before. Orange color marks the distal, non-myocardial portion of the embryonic outflow tract.

**Figure 6:**
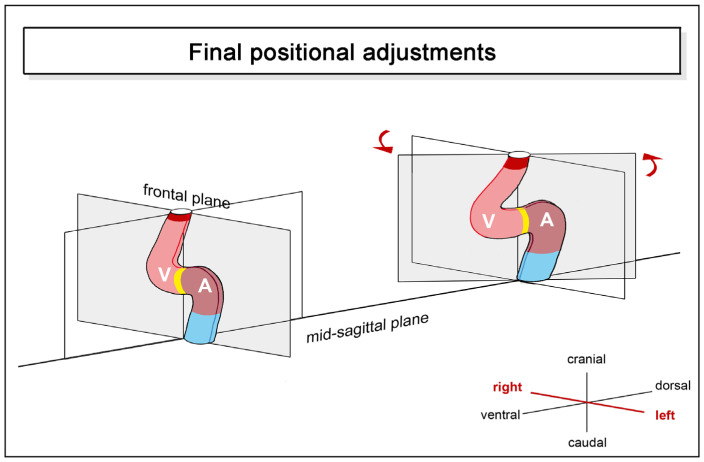
This schematic drawing illustrates the final positional and morphological changes of the looping embryonic heart tube along the left-right body axis as seen in teleost fishes. These changes may be described as back-rotation around the longitudinal heart axis. Color codes and abbreviations as used before.

The ‘S-shaped’ heart loop of these animals shows only slight twisting around the atrioventricular canal [19] so that these hearts appear as a plane S rather than a three-dimensional geometrical object [18,23] (Figure 1(A1) and Figure 4A). In the second group (hearts of cartilaginous fishes, etc.), only the ventricular portion and its myocardial outflow element become lateralized, whereas the atrial portion of the heart loop does not undergo rightward rotation (Figure 4B). The looping hearts of these animals, thus, are subjected to considerable torsion so that their ‘S-shaped’ configurations tend to resemble helically wound tubes rather than plane geometrical objects. Moreover, due to the fact that the myocardial outflow element becomes displaced to the right of the body together with the ventricular bend, the straight outflow element changes its axial orientation from an initially vertical to a transverse axis (Figure 5(B1)). The most complex form of helical heart looping is found in lungfish and tetrapods. The embryonic heart loops of these animals not only develop a counterclockwise helical winding of the ventricular bend and its inflow portion but also develop a clockwise helical winding of the ventricular outflow element [41] (Figure 1(B, No. 1) and Figure 5(B, No. 2)). The latter feature may be of special interest since it may set the scene for the development of the clockwise ‘spiral’ course of the great arteries found in the mature heart of higher vertebrates [42].

(4), **final positional adjustments**: the positional changes characterizing this component may be described as a kind of back-rotation or untwisting of the heart loop, which leads to a reduction in the degree of lateral/chiral looping reached at earlier stages of cardiogenesis (Figure 6).

**Figure 7:**
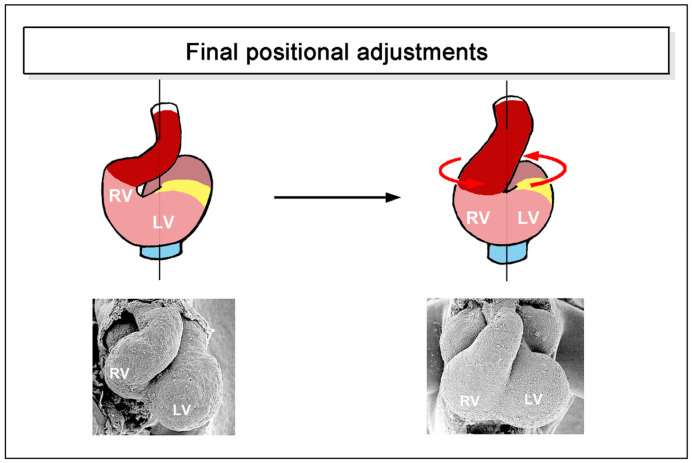
These schematic drawings and scanning electron microscopic images illustrate the process of untwisting the heart loop, as seen in higher vertebrates (scanning electron microscopic images depict mouse hearts at embryonic day 9.5 and 11). Hearts are shown in frontal views. For a more detailed discussion of heart loop untwisting in higher vertebrates, see [34]. Abbreviations: RV = embryonic right ventricle; LV = embryonic left ventricle. Color code as used before.

Untwisting of the heart loop was first observed in teleost fish (*Cyprianus blicca*) by the Baltic German scientist Karl Ernst von Baer in 1835 [1]. Compared to lateral/chiral looping, however, this looping component has not received much attention. It may be no wonder, therefore, that it was later rediscovered several times in diverse teleosts [7,44,45,46]. Untwisting of the developing heart has been observed not only in modern ray-finned fishes but also in lungfish [9] and higher vertebrates [6,34,47] (Figure 7).

In teleost fishes, untwisting of the developing heart is observed at the transition between the embryonic and larval periods of development [44,45,46], while in higher vertebrates it occurs at embryonic stages [6,34]. At the end of untwisting, the developing heart chambers and great vessels have normally reached an approximation of their definitive topographical relationships.

## 3. Comparative Anatomy of the Looped Design of Mature Vertebrate Hearts

Comparative anatomical data reveal some striking species- or class-specific differences in the situs of mature vertebrate hearts. These differences may be explained by differences in the extent to which the above-described looping components contribute to the definitive positioning of the heart chambers. The present comparative analysis of the definitive heart situs of vertebrates will focus mainly on two aspects. These aspects are, firstly, those positional relationships of the atrial and ventricular chambers that primarily cause the ‘S-shaped’ (sigmoid) configuration of the main course of the cardiac flow path(s), and, secondly, those anatomical features that cause a bilaterally asymmetric morphology of the hearts responsible for twisting of the sigmoid cardiac flow path(s). The first aspect may be named the “sigmoid routing of the cardiac flow path(s)”. Its status mainly reflects the extent to which the dorsoventral and craniocaudal looping components contribute to the definitive heart situs. The second aspect may be named the “bilaterally asymmetric routing” or “chiral routing” of the cardiac flow path(s). Its status mainly reflects the extent to which lateral/chiral looping and final positional adjustments (untwisting) contribute to the definitive heart situs.

### 3.1. Comparative Anatomy of the Sigmoid Routing of the Flow Path(s) of Mature Vertebrate Hearts

Sigmoid routing of the cardiac flow path(s) was found in the mature heart of all extant vertebrates studied so far [24,30,39,40]. Sigmoid routing of the cardiac blood stream(s), therefore, is a phylogenetically highly conserved feature among vertebrates. The degree of sigmoid routing, however, was found to differ considerably between the species examined. In contemporary articles on the evolution of the vertebrate heart, the degree of sigmoid routing of the cardiac flow path(s) is frequently considered to reflect the position of the species along the phylogenetic tree of vertebrates [30,39,40]. Thereby, the hearts of basal vertebrates (jawless fishes) are shown with the lowest degree of sigmoid routing, mainly resulting from a ‘primitive’ atrial position that is dorso-caudal to the ventricle. The ventricular flow path of these hearts makes a roughly 45° turn from the atrioventricular to the arterial valve (ventricular J-turn loop). The hearts of jawed fish are shown to have a moderate degree of sigmoid routing, resulting from an atrial position that is dorsal to the ventricle. The ventricular flow path of these hearts makes a roughly 90° turn from the atrioventricular to the arterial valve (ventricular L-turn loop). The hearts of tetrapods (amphibia, reptiles, birds, and mammals) usually have the highest degrees of sigmoid routing, resulting from an atrial position that is dorso-cranial to the ventricle(s). The ventricular flow paths of these hearts make roughly 120° to 150° turns from the atrioventricular to the arterial valves (ventricular U-turn loop).

**Figure 8:**
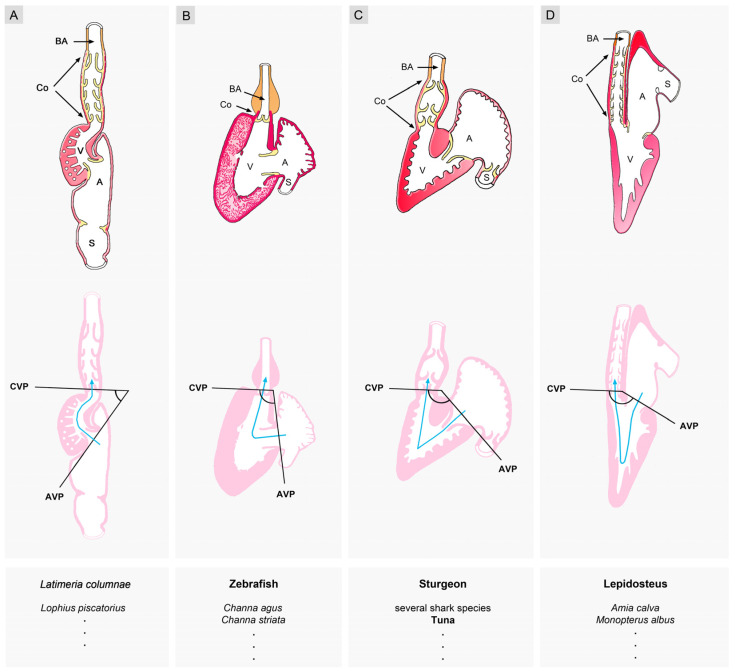
These semi-schematic drawings illustrate the variation in the degree of sigmoid routing of the cardiac flow path found among the two-chambered hearts of jawed fish. The spectrum ranges from low degrees (**A**) to very high degrees of sigmoid routing (**D**). The situation in the latter heart resembles the situation found in the left heart of higher vertebrates, such as human beings. The drawings depict hearts from (**A**) *Latimeria columnae*, (**B**) zebrafish, (**C**) sturgeon, and (**D**) gar as examples of low, moderate, high, and very high degrees of sigmoid routing of the cardiac flow path, respectively (ventricular flow paths are shown by curved blue arrows). Additional fish species with a similar degree of sigmoid routing of the cardiac flow path are listed in the columns below each example. The angle between the atrioventricular plane (AVP) and the cono-ventricular plane (CVP) can be used as a measure of the degree of atrial ascent. Note that the degree of atrial ascent does not reflect the position of the species along the phylogenetic tree of vertebrates. Drawings are based on image data from [53,54] (**A**), own histological specimens (**B**), [55] (**C**), and [56] (**D**). Abbreviations: BA = bulbus arteriosus; Co = conus arteriosus; S = sinus venosus; other abbreviations as used before.

It should be noted here, however, that a critical review of previously published original data shows that the above-described phylogenetic scenario does not completely match reality. With regard to basal vertebrates (jawless fishes), we can note that the atrium of the mature hagfish heart indeed lies in the above-described ‘primitive’ position [48,49,50]. The atrium of the mature heart of lampreys, however, is found at the same craniocaudal level as the ventricle [49,51,52], which is the position usually assigned to jawed fish. The atrium of the mature heart of jawed fish, on the other hand, is not regularly found at the same cranio-caudal level as the ventricle. Among jawed fish species, we can rather find a spectrum of various degrees of sigmoid routing of the main cardiac blood stream (Figure 8).

**Figure 9:**
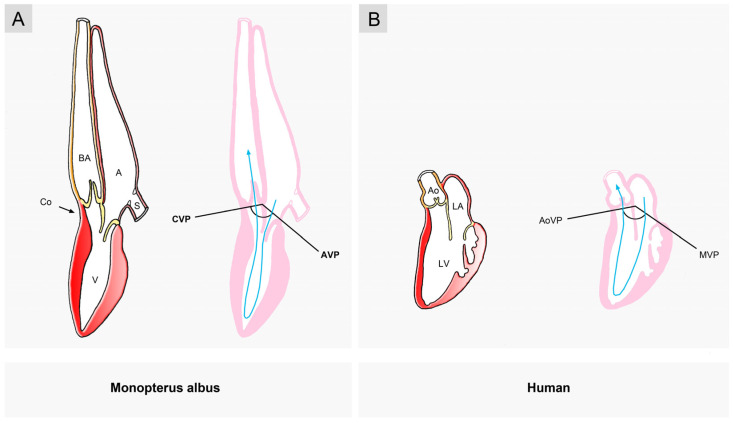
These semi-schematic drawings illustrate that some fish species have hearts with a high degree of sigmoid routing of the cardiac flow path corresponding to the situation found in the left heart of higher vertebrates, such as human beings. The drawings depict the hearts of *Monopterus albus* (**A**) and humans (**B**). Drawings are based on image data from [58,59] (**A**) and [60] (**B**). Abbreviations: Ao = ascending aorta; AoVP = aortic valve plane; LA = left atrium; LV = left ventricle; MVP = mitral valve plane; other abbreviations as used before.

This spectrum ranges from low degrees, resulting from an atrial position that is dorso-caudal to the ventricle (Figure 8A) (e.g., coelacanths, see [53]; *Lophius piscatorius*, see [57]), to high degrees, resulting from an atrial position that is dorso-cranial to the ventricle (Figure 8D) (e.g., lepisosteiformis, amiiformis, see [56]; *Monopterus albus*, see [58,59]). Thus, a high degree of sigmoid routing of the main cardiac bloodstream(s) is not exclusively observed in the mature hearts of tetrapods. It can also occur in the hearts of some fish species (Figure 9).

In contrast to the high range of variation in the extent of sigmoid routing of the cardiac blood streams found in fish, I could not find any report on tetrapod hearts with a low or moderate degree of sigmoid routing of the cardiac flow path(s). Thus, a high degree of sigmoid routing of the cardiac blood streams seems to be a common feature of the mature heart of tetrapods.

### 3.2. Comparative Anatomy of the Bilaterally Asymmetric (Chiral) Routing of the Flow Path(s) of Mature Vertebrate Hearts

**Figure 10:**
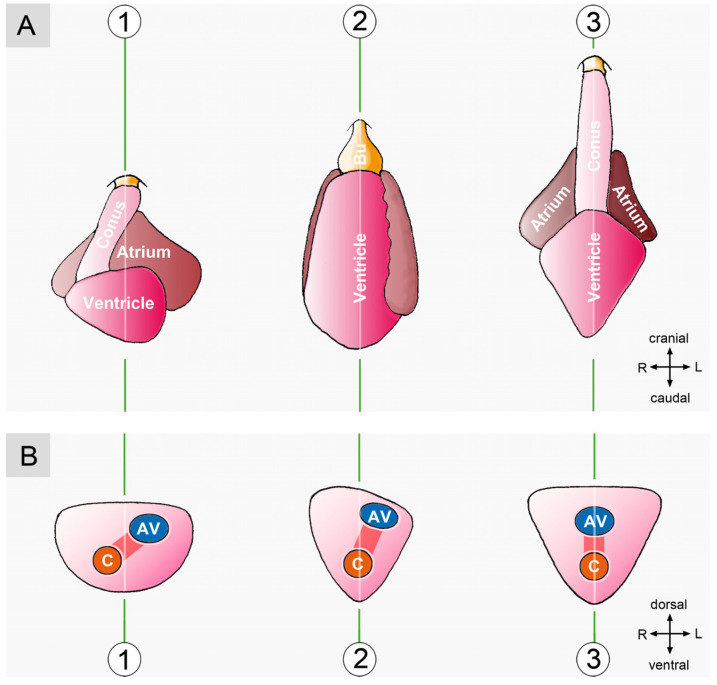
These semi-schematic drawings illustrate the grouping of fish hearts into three different morphological groups according to the degree of bilateral asymmetry/symmetry: (1) hearts of visually conspicuous asymmetry (**A**, No. 1; **B**, No. 1); (2) hearts of visually obscured asymmetry (**A**, No. 2; **B**, No. 2); and (3) hearts of nearly perfect bilateral symmetry (**A**, No. 3; **B**, No. 3). Panel (**A**) depicts the hearts in frontal views, while panel (**B**) depicts views on the base of the ventricles of the corresponding hearts. The drawings depict hearts from (1) cartilaginous fish (ray), (2) zebrafish, and (3) gar as examples for the respective group. AV marks the mouth of the atrioventricular canal; C marks the entrance into the conus arteriosus. The midsagittal body plane is marked with green lines. Drawings are based on image data from [56,66,67,68] (**A**, No. 1; **B**, No. 1; **A**, No. 3; **B**, No. 3) and own specimens (**A**, No. 2; **B**, No. 2).

It is generally stated in the biomedical literature that the inner organs of vertebrates display a bilaterally asymmetric anatomy and that the heart is the first inner organ of vertebrates to adopt a bilaterally asymmetric shape during ontogenesis [61,62,63]. Moreover, it is a well-known fact that the bilaterally asymmetric anatomy of the human heart provides the basis for the structural division of its lumen into pulmonary and systemic flow paths. It, therefore, may be no wonder that the mature heart of vertebrates is generally seen as an organ of bilaterally asymmetric anatomy [62,64,65]. While this view is valid for the hearts of tetrapods, the situation in fish does not match with it. Based on the degree of bilateral asymmetry, the morphological phenotypes of fish hearts can be roughly assigned to three different groups: (1) hearts with a visually conspicuous bilateral asymmetry, (2) hearts with a visually obscured bilateral asymmetry, and (3) hearts with a nearly perfect bilateral symmetry (Figure 10).

Bilaterally asymmetric routing of the cardiac flow path can frequently be noted during the first visual examination of the outer shape of a fish heart. This is relatively easy in those fish hearts that have a relatively long myocardial outflow element called the *conus arteriosus*. The conus arteriosus connects the ventricle to the *bulbus arteriosus*, which is the intrapericardial root of the ventral aorta. The bulbus arteriosus and the ventral aorta are normally aligned in the midsagittal body plane. Alignment of the conus arteriosus in the midsagittal body plane, together with the bulbus arteriosus and the ventral aorta, is highly suggestive of the presence of bilateral symmetry (Figure 10(A, No. 3)). Deviations of the long axis of the conus arteriosus from the midsagittal body plane, on the other hand, indicate bilaterally asymmetric routing of the cardiac flow path (Figure 10(A, No. 1)).

While analyzing previously published data on the chirality of fish hearts, I have noted that there seems to be a striking association between the degree of bilateral cardiac asymmetry and the body shape of fish. Pronounced left-right asymmetry of the heart is frequently found in fishes that show flattening of their body along the dorsoventral body axis, such as rays and angel sharks. Hearts with nearly perfect bilateral symmetry, on the other hand, are frequently found in fishes whose bodies have a fusiform cross-section, such as tunas and gars. It is conceivable that a dorso-ventrally flattened body limits the dorsoventral dimensions of the pericardial cavity but supports lateral (left-right) expansion of the pericardial cavity. The establishment of such a spatial condition during the larval stages of development may have forced the post-embryonic heart to retain its embryonic state of bilateral asymmetry. A fusiform body cross-section, on the other hand, may limit the lateral (left-right) dimensions of the pericardial cavity but supports the dorsoventral expansion of the pericardial cavity. The establishment of such a spatial condition during the larval stages of development may have forced the post-embryonic heart to rotate back to its original orientation along the dorsoventral body axis and thereby lose its state of bilateral asymmetry reached at the end of the embryonic period of development. At first sight, this idea appears to be very speculative. It should be noted here, however, that some previous researchers have already found that the shape of the ventricle of a teleost heart reflects the shape of the body of the fish [69,70]. Future studies are needed to clarify the relationship between body shape and cardiac morphology in embryonic as well as post-embryonic fishes.

#### 3.2.1. Hearts with a Visually Conspicuous Bilateral Asymmetry

**Figure 11:**
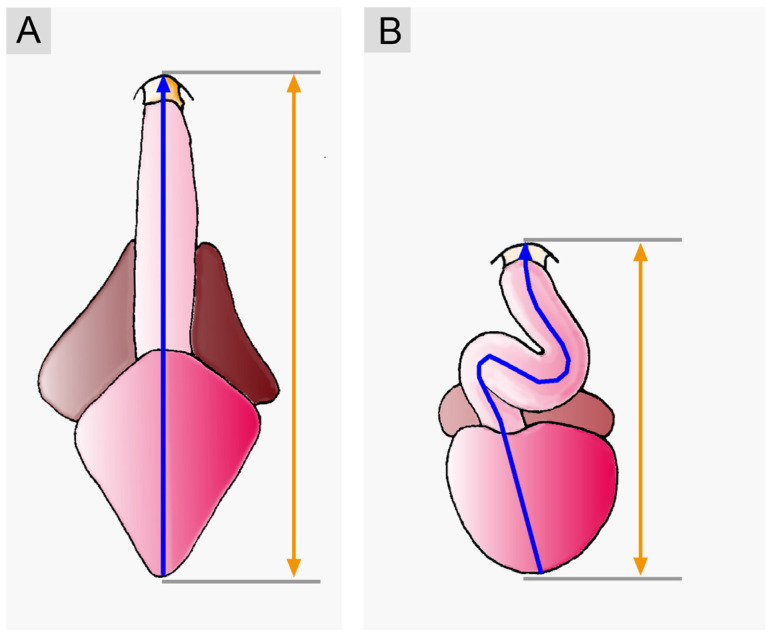
These drawings depict the bilaterally symmetric heart of a gar (**A**) and the bilaterally asymmetric heart of a lungfish (**B**). Note that in the gar, the greatest length of the pericardial cavity (orange arrow) corresponds to the length of the systolic ventricular flow path (blue arrow). In lungfish, however, there is a striking discrepancy between the greatest length of the pericardial cavity and the length of the systolic ventricular flow path. Hearts are shown at the same relative size. Based on the image data from [56,68] (**A**) and [73] (**B**).

The hearts of cartilaginous fish and some ‘primitive’ ray-finned fish, such as sturgeons, show conspicuous bilateral asymmetry (Figure 10(A, No. 1)). The conus arteriosus of these hearts is a relatively long and straight myocardial tube that originates from the right side of the ventricle and runs in an oblique course to the bulbus arteriosus. Moreover, the atrioventricular valve opens into the left portion of the ventricle so that the ventricular inflow and outflow orifices are arranged laterally to one another (Figure 10(B, No. 1)) [67,71,72]. The ventricle of such hearts, therefore, was described as displaying a “looped appearance” [71]. Asymmetric routing of the ventricular flow path can become apparent not only by a lateral displacement of the origin of a long conus arteriosus but also by a helical deformation of its shape. This situation has been observed in lungfish [56,73] and is associated with a striking discrepancy between the length of the systolic ventricular flow path and the greatest length of the pericardial cavity (Figure 11).

Apart from an asymmetric arrangement of the ventricle and its outflow tract, a visually conspicuous asymmetry of a mature fish heart can also be characterized by primitive left-right positioning of the two heart chambers. In jawless fish, for example, the atrium is frequently said to lie to the left of the ventricle [50,51,52]. The mature hearts of these fishes seem to retain their maximum degree of embryonic heart loop lateralization during the entire post-embryonic lifespan of the animals. This suggests that the fourth component of cardiac looping (back-rotation/untwisting) does not significantly contribute to the mature cardiac phenotype in these species. I should note here, however, that there exists some conflicting data about the final left-right positioning of the heart chambers of jawless fish. Some authors have reported that the left-sided atrium of the embryonic lamprey heart becomes placed more dorsally to the ventricle at later stages of development [74,75], and another group has reported that the mature hagfish heart occupies a midline position in the body and that its atrium lies dorsal and caudal to the ventricle [48]. These findings are at variance with the above-mentioned observations and would suggest that the developing heart of jawless fishes may undergo untwisting. Future studies are needed to clarify this situation.

#### 3.2.2. Hearts with a Visually Obscured Bilateral Asymmetry

‘Modern’ ray-finned fishes (teleosts) have only a very short conus arteriosus [76,77,78]. Due to its small length, the axis of this ring-shaped outflow element is always aligned in the midsagittal body plane, where it connects the ventricle with the bulbus arteriosus (Figure 10(A, No. 2)). Such a situation can obscure the presence of asymmetric routing of the cardiac flow path if the heart does not display additional, externally visible signs of bilateral asymmetry, such as a left-sided position of the atrium (see above). It, therefore, may be no wonder that the hearts of teleost fishes have frequently been described as bilaterally symmetric structures [49,67,71]. Analysis of the internal anatomy of such hearts, however, frequently discloses an asymmetric position of the central portion of the atrium dorsal and to the left of the ventricle in combination with a left-sided position of the atrioventricular orifice (Figure 10(B, No. 2)) [79,80,81].

#### 3.2.3. Hearts with a Nearly Perfect Bilateral Symmetry

In several species of ray-finned fish (holostei, e.g., lepisosteiformis, amiiformis, as well as teleostei, e.g., tuna), the mature heart occupies a midline position in the body of the animals and appears as a nearly perfect, bilaterally symmetric structure (Figure 10(A, No. 3)). In these hearts, the atrium lies dorsal or dorso-cranial to the ventricle and atrioventricular canal, ventricular outflow tract, and ventral aorta, all of which are aligned in the midsagittal body plane (Figure 10(B, No. 3)) [56,67,82]. We have no embryological data from lepisosteus or tuna fishes that can answer the question of whether the nearly perfect symmetry of their mature hearts results from a physiological lack of lateral/chiral looping of their embryonic hearts or from the complete elimination of an already adopted bilaterally asymmetric shape during the process of final positional adjustments. Embryological data from *Amia calva*, however, show that the embryonic hearts of this fish species undergo S-looping as well as lateral/chiral looping in the same way as in other fishes [83]. This finding suggests that mature fish hearts with nearly perfect bilateral symmetry have completely lost their embryonic state of lateral/chiral looping while they have retained their embryonic state of dorsoventral and craniocaudal looping. It is likely that the loss of bilateral asymmetry is accomplished by complete back-rotation of the developing heart during the process of final positional adjustments. In these fish, the existence of a bilaterally asymmetric heart seems to be a transitory phenomenon confined only to the embryonic period of development.

## 4. The Functional Significance of the Looped Design of Embryonic and Mature Vertebrate Hearts

If we want to clarify the functional significance of the design of an inner organ or a technical device, we should consider at least three different possibilities: (1) a certain feature of the design may make important contributions to the specific function(s) of the organ/technical device under investigation. (2) some aspects of the positioning and shaping of an organ/technical device may not directly contribute to its specific function(s) but merely represent functional solutions to other demands, e.g., a packing problem or the protection of the organ against physical damage. (3) some organs or some of their form features may not have any function at the time point of investigation. They may actually represent functionless vestiges from earlier phases of individual life or from earlier phases of phylogeny.

Functional interpretations of the looped design of vertebrate hearts have generally focused on their possible roles in the pumping function of the heart. The possibility that a given feature of the looped design may represent a solution to other functional demands of the body, such as a packing problem, or may represent a functionless vestige from earlier stages of ontogenesis or phylogenesis, has largely been ignored by the scientific community. The diverse concepts about the functional consequences of cardiac looping can roughly be assigned to two different functional aspects: (1) the determination of the definitive spatial arrangement of the building units of the heart (chambers, connecting elements, and great blood vessels), which dictates the alignments and separations of the systemic and pulmonary flow paths within the multi-chambered hearts of tetrapods; and (2) the optimization of the cardiac pumping efficiency.

### 4.1. A Closer View on the Functional Design of Embryonic Vertebrate Hearts

In the past, concepts regarding the functional significance of the looped design of vertebrate hearts usually focused on the mature heart. The possibility that cardiac looping might have any effect on the pumping function of the valveless embryonic heart tube has been ignored by most developmental biologists since it was thought that the looped design of the vertebrate embryonic heart tube has functional consequences only at advanced stages of development when the heart has acquired the mature phenotype of a chambered heart [84]. The long-lasting prevalence of the paradigm of late-onset effects of cardiac looping is astonishing since the looped heart tube shows several form features (e.g., s-shaped curvatures, kinking, helical coiling, torsion) that are known for their potential to significantly change the hemodynamics of circulatory systems at macro-vascular as well as micro-vascular scales (reviewed by [27]). Moreover, the appearance of these features during embryonic cardiogenesis coincides with the onset of hemodynamically effective blood circulation [6,85,86], and data from zebrafish mutants suggest that failure of heart looping can reduce the pumping efficiency of embryonic hearts [87,88]. Based on these and other arguments, my group has postulated that the looped design of the tubular heart of vertebrate embryos might improve the efficiency of valveless pumping. The physical plausibility of our hypothesis was tested on a valveless pump model in which unidirectional fluid flow was generated by the so-called Liebau effect, which is a pumping mechanism suspected to generate blood flow in the vertebrate embryonic heart tube [89]. It was found that, under the same conditions of actuation, a looped configuration of our tubular pump generated higher maximum pressure heads and higher average flow rates than the straight configuration [27]. These data principally confirmed the physical plausibility of our hypothesis. Unfortunately, however, we could not identify the mechanism responsible for the improvement in pumping efficiency. It might be possible that the kinks in our valveless heart loop model, which are typical features of the looped heart tube of vertebrate embryos, have acted in a valve-like manner. Furthermore, I have to note that at the present time, it is unclear as to whether the valveless heart tubes of vertebrate embryos generate unidirectional blood flow via the above-mentioned Liebau effect or via peristalsis [31]. Thus, our initial simulation experiments should be complemented by future studies aimed at clarifying whether a looped design might also improve the efficiency of peristaltic pumps. In view of our initial data from physical models, however, I think that we should no longer ignore the possibility that looping morphogenesis might optimize the pumping function of the valveless heart tube of vertebrate embryos. Moreover, if we assume that the mature heart of vertebrates has phylogenetically evolved from the tubular heart of a vertebrate ancestor [90], similar to the situation found in extant urochordates such as *Ciona intestinalis*, we can postulate that the phylogenetically oldest function of cardiac looping might have been the improvement of the pumping efficiency of the tubular heart of vertebrate ancestors.

### 4.2. A Closer View on the Functional Design of Mature Vertebrate Hearts

To understand the functional significance of the looped design of mature vertebrate hearts, it is helpful to roughly distinguish between two different groups of hearts: the two-chambered hearts and the multi-chambered hearts. Two-chambered hearts represent the basic design of the mature vertebrate heart. They are found in all groups of fish, with the exception of lungfish. Two-chambered hearts consist of a single atrial chamber, which receives the blood from a confluence of veins called the sinus venosus, and a single ventricular chamber, which is connected with the atrium via the so-called atrioventricular canal and pumps the blood into the ventral aorta via ventricular-aortic connecting elements (conus arteriosus, bulbus arteriosus). The two-chambered hearts of vertebrates have only a single intra-cardiac flow path, which transports either deoxygenated (gill-breathing fishes) or mixed blood (air-breathing fishes) [91]. The group of multi-chambered hearts comprises three- (two atria and one ventricle) and four-chambered (two atria and two ventricles) hearts. The common feature of these hearts is the presence of two intra-cardiac flow paths, which are functionally or structurally separated from each other. One path directs the oxygenated blood from the lungs into the systemic circuit, and the other directs the deoxygenated blood from the systemic veins into the pulmonary circuit. Multi-chambered hearts have evolved from two-chambered hearts during vertebrate evolution and, therefore, represent phylogenetically younger variants of vertebrate heart design. They are found in lungfish and tetrapods. The proper function of such hearts depends on hemodynamically or structurally correct alignments and the separation of their intra-cardiac flow paths.

#### 4.2.1. Looping Dictates the Alignments and Separations of the Systemic and Pulmonary Flow Paths

**Figure 12:**
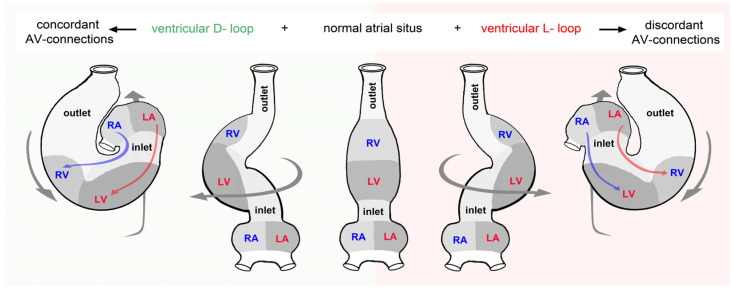
This scheme illustrates the importance of cardiac looping, especially chiral looping, for the alignment of flow paths in the multi-chambered hearts of higher vertebrates. In cases of normal left-right patterning of the atrial chambers, rightward rotation of the ventricular bend (ventricular D-loop) sets the scene for correct (concordant) alignment between the atrial and ventricular heart chambers (green background). However, the combination of normal left-right patterning of the atrial chambers with leftward rotation of the ventricular bend (ventricular L-loop) can set the scene for incorrect (discordant) alignment between the atrial and ventricular heart chambers (red background). Abbreviations: AV = atrioventricular; LA = future left atrium; LV = future left ventricle; RA = future right atrium; RV = future right ventricle.

Since the early 20th century, the major function of embryonic heart looping has been to dictate the future alignment and separation of the pulmonary and systemic flow paths within the mature, multi-chambered heart [34,35,47,63,92,93,94]. In this context, the lateral/chiral component of cardiac looping is especially seen as essential for the determination of the atrioventricular as well as ventriculoatrial connections (Figure 12).

The strong and long-lasting focus on this functional consequence of cardiac looping may be explained by a wealth of evidence indicating that abnormal looping of the embryonic heart, especially disturbances in lateral/chiral looping, plays a central role in the pathogenesis of complex congenital heart defects with abnormal alignments and separations of the cardiac flow paths [95,96,97,98,99]. A discussion of the relationship between abnormal looping and phenotypes of congenital heart defects is very interesting but would go beyond the scope of the present review article. Readers who are especially interested in this topic are thus referred to previous reviews dealing with this topic [23,34,99].

**Figure 13:**
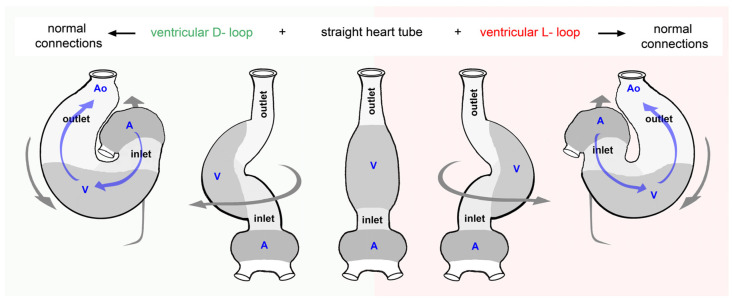
This scheme illustrates the fact that cardiac looping does not determine the atrioventricular or ventriculoatrial alignment in the two-chambered hearts of fishes. Abbreviations and color codes as used before.

The previous focus on the role of chiral looping in the development of higher vertebrate hearts may have hampered the perception of the fact that the above-described functional interpretation of cardiac looping can be valid only for the hearts of tetrapods and lungfish. The two-chambered hearts of fishes have only a single undivided flow path, and their chambers are normally properly connected with each other and with the arterial and venous branches of the circulation before the embryonic heart starts its looping [100]. Dictating the future alignments and separations of the pulmonary and systemic flow paths within the mature, multi-chambered heart of vertebrates, therefore, cannot be regarded as a phylogenetically conserved function among vertebrates (Figure 13) but merely should be regarded only as a phylogenetically relatively young function of cardiac looping. Therefore, the question is: what are the phylogenetically oldest functions of the looped heart design that are conserved in the two-chambered hearts of fishes as well as in the multi-chambered hearts of lungfish and tetrapods? The answer should be found in the group of concepts that examines the functional consequences of the looping process in the optimization of the pumping performance of the mature vertebrate heart.

#### 4.2.2. Looping Might Optimize the Cardiac Pumping Efficiency

Up to the end of the 20th century, several researchers had speculated that the looped design of the vertebrate heart might improve its pumping function. Unfortunately, however, none of these researchers had presented a physically sound concept about the way in which a looped configuration might improve cardiac pumping function. The scene changed in 2000 when Philip Kilner and co-authors presented, for the first time, physically sound concepts that were based on magnetic resonance phase-velocity mapping of the blood flow patterns in the human heart [24,25,101]. During the past 25 years, Kilner’s concepts have been supplemented by further physically sound hypotheses [26,28] so that, at the present time, we can distinguish between five postulated benefits for the pumping function of vertebrate hearts. Of these five advantages, only three may be valid in the mature hearts of fish as well as tetrapods, while two advantages seem to be valid only in tetrapods. The description of the five postulated advantages thus starts with the latter two advantages that were published by the group of Bruno Marino [26] and the group of Mark Sherrid [28].

Marino and co-workers have noted that lateral/chiral looping does not only seem to dictate the future atrioventricular and ventriculo-arterial connections of the mature heart of tetrapods. It, furthermore, causes the ‘spiral’ (helical) arrangement of the ventricular outflow tracts and arteries normally found in the mature heart of higher vertebrates. They speculated that this chiral form feature may produce a hemodynamically advantageous situation. Computational fluid dynamics simulation and three-dimensional flow imaging indeed have shown that the ‘spiral’ (helical) pattern of the great arteries has fluid dynamic advantages compared to a non-‘spiral’ (linear) pattern typically found in congenital heart defects with transposition of the great arteries [26]. Since the two-chambered hearts of fishes do not show a ‘spiraling’ of their outflow tracts, this functional benefit of cardiac looping cannot be regarded as a phylogenetically old function.

Sherrid and co-workers focused on the functional significance of the sigmoid routing of the cardiac flow path(s). They were especially interested in identifying the possible advantages of the ventricular U-turn loop of the human heart, which resulted from the atrial ascent/ventricular descent during cardiac looping. They have hypothesized that the craniodorsal position of the atria, which is typical for the multi-chambered hearts of humans and other tetrapods, may provide a favorable hydrostatic position for systemic venous return as well as pulmonary venous return in land-living vertebrates in which the effects of gravity on the circulating blood are much higher than in water-living fishes [28]. Since the effects of gravity on circulating blood are largely canceled out in water [102], the craniodorsal position of the atrium found in the two-chambered heart of some fish species (see above) can hardly be interpreted in the same way as in land-living vertebrates. Thus, we speculate that this feature of the looped design of mature vertebrate hearts may provide more than a single functional advantage. A hydrostatically favorable position may then be regarded as a second, phylogenetically younger functional advantage of the craniodorsal position of the atrium, which became functional only when the ancestors of land-living vertebrates left their original aquatic habitat. The Asian swamp eel (*Monopterus albus*) can be regarded as a model for such a scenario. This teleost fish has a two-chambered heart in which the atrium lies in a craniodorsal position [58,59]. When this eel-like fish is held outside of the water, in a vertical position with its head upwards, its beating heart is continuously refilled with blood. The beating heart of a true eel (*Anguilla anguilla*), however, in which the atrium lies dorsal to the ventricle, will rapidly become empty when the fish is held in the same position [58].

Before I present and discuss the remaining three postulated advantages of a looped heart design, which may be valid for the pumping function of multi-chambered hearts as well as two-chambered hearts, I should point to one previously neglected but functionally important difference between the two groups of vertebrate hearts. As noted in the section on comparative anatomy, multi-chambered hearts regularly show a bilaterally asymmetric (chiral) anatomy. The functional implications of this feature were discussed in the preceding paragraphs. Two-chambered hearts, on the other hand, do not regularly show a bilaterally asymmetric configuration (Figure 10). Among fish, we can find species with bilaterally asymmetric hearts and species with bilaterally symmetric hearts. The presence of a nearly perfect bilateral symmetry, especially in the high-performance hearts of tuna [103] and other active fishes with a predatory habit, suggests that chiral routing of the cardiac flow path may not significantly contribute to an improvement of the pumping function of two-chambered hearts. Therefore, if we believe that the pumping function of two-chambered hearts may profit from a looped design, such a benefit should be attributed to the sigmoid routing of the cardiac flow path, which is a feature found in the two-chambered and multi-chambered hearts of vertebrates. The questions then arise: (1) what are the functional implications of chiral looping in fishes? and (2) how can sigmoid routing of the cardiac flow path(s) improve the pumping function of two-chambered as well as multi-chambered hearts?

With regard to the first question, I should refer to the above-mentioned observation that a bilaterally asymmetric heart shape frequently is found in fishes with a dorso-ventrally flattened body shape while a bilaterally symmetric heart frequently is found in fishes with a laterally ‘compressed’ body shape (e.g., tuna). This finding suggests that a bilaterally asymmetric arrangement of the building units of a two-chambered heart may represent no more than the solution to the problem of packing an s-shaped heart into a body of narrow ventro-dorsal dimensions but wide lateral dimensions. The variability in the positioning of a two-chambered heart within the body of fish may be compared with the variability in the mounting of an engine into a vehicle, which can be made transverse to the long axis or along the long axis of a car. A striking limitation of the ventrodorsal dimension of the pericardial cavity is found not only in some fishes but also in early vertebrate embryos, where it is regarded as one of the mechanical factors responsible for the chiral/lateral looping of the embryonic heart tube [6]. We, therefore, may speculate that, during the evolution of vertebrates, the chiral component of the looped design of their hearts originally may have represented no more than the solution to the problem of packing the embryonic as well as mature s-shaped heart into a physically confined space. The evolution of the genetic control of the left-right patterning of the inner organs of vertebrates may then be primarily interpreted in terms of optimal packing.

With regard to the second question, I can refer to the functional concepts published by Kilner and co-authors [24,25,101]. Based on imaging of the blood flow patterns in the human heart, this group has postulated that, compared to a heart with a linear routing of its flow path, the sigmoid routing of the flow path(s) of vertebrate hearts may confer three interrelated advantages, which are thought to gain significance during strenuous exertion. (1) **stabilization of flow**. Compared to linear routing, sigmoid routing of the flow path(s) causes a more eccentric (tangential) streaming of the inflowing blood, which may allow more stable, less turbulent filling of the heart cavities, thereby reducing the dissipation of energy through turbulence. (2) **minimizing the loss of momentum of the inflowing blood**. Sigmoid routing of the flow path(s) causes eccentric recirculation of the inflowing blood at both atrial and ventricular levels that redirects blood preferentially toward the next chamber or outflow vessel. This may minimize the loss of momentum so that the contraction of a chamber only serves to add energy to the blood already moving toward the next chamber (ventricle) or to the arterial stem vessel(s). In a linear heart with a centrally directed flow, the inflow is thought to result in greater instability and turbulence and redirects the inflowing blood away from the next chamber or vessel. (3) **enhancement of ventriculoatrial coupling**. In a two-chambered heart with a linear arrangement of its building units, such as the heart of the snail *Helix pomatia*, vigorous ejection of blood during ventricular systole is thought to produce a recoil of the ventricle, similar to a gun, away from the direction in which the blood is ejected. This is thought to inhibit the reciprocating long-axis displacement of the atrio-ventricular plane (AVP) during the cardiac cycle, which plays an important role in the refilling of the atrial and ventricular chambers of the heart of higher vertebrates [60,104,105,106,107]. Only in the vertebrate heart, with a ventricular U-turn loop, is the postulated systolic recoil of the ventricle thought to be directed to enhance rather than inhibit long-axis displacement of the AVP, thus supporting atrial filling during the ventricular systole, particularly in the exercising state.

#### 4.2.3. A Critical Evaluation of the Proposed Functional Advantages of the Sigmoid Routing of the Cardiac Flow Path(s)

The above-mentioned concepts provide physically sound descriptions of postulated mechanisms by which sigmoid routing of the flow path(s) of mature vertebrate hearts might optimize the cardiac pumping function. So far, however, two basic questions remain unanswered: (1) do these mechanisms really work in vertebrate hearts, and, if so, (2) do these mechanisms really lead to a significant improvement of the pumping function of a looped as opposed to a linear heart?

##### Does Looping Really Minimize the Loss of Momentum of the Inflowing Blood?

Since the first publication of Kilner’s concepts in 2000, only a single study was conducted in which the validity of one of the three postulated advantages was tested by the use of finite element numerical modeling of the hemodynamics of virtual left ventricles [108]. The aim of this study was to compare the hemodynamics of the left ventricles with physiologically directed inflow paths (toward the apex) and non-physiologically directed inflow paths (toward the interventricular septum). It has been reported that the physiological flow path does not have an energy-saving effect. These data seem to suggest that sigmoid routing of the cardiac flow path(s) does not improve the efficiency of cardiac pumping by minimizing the loss of momentum of the inflowing blood. In a comment on this paper, however, Kilner noted that the above-mentioned numerical modeling study did not compare the hemodynamics of virtual hearts with a looped and non-looped (linear) arrangement [109]. Thus, answering the two above-mentioned questions awaits the results of functional studies in which the pumping function of real or virtual hearts with a looped design is compared with the pumping function of non-looped (linear) hearts.

##### Does Looping Really Enhance Ventriculoatrial Coupling?

**Figure 14:**
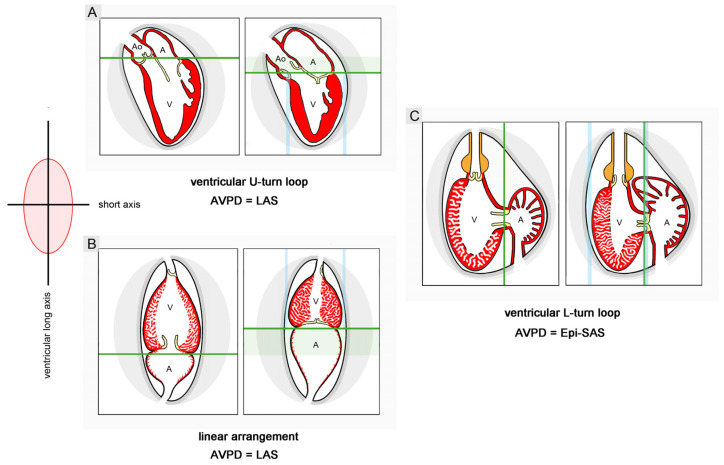
These drawings depict the relationship between the arrangement of the heart chambers and the atrioventricular plane displacement (AVPD) during the cardiac cycle. An appreciable AVPD (indicated by transparent green bands) occurs in hearts in which the AVP (marked by green lines) lies perpendicular or almost perpendicular to the ventricular long-axis. Such situations are found in hearts with a very high degree of sigmoid routing of the flow path (ventricular U-turn loop), such as the left heart of human beings (**A**), or in hearts with a linear arrangement of its chambers, such as the heart of the snail *Helix pomatia* (**B**). In hearts with a moderate degree of sigmoid routing (ventricular L-turn loop), such as the zebrafish heart (**C**), the AVP lies perpendicular to the ventricular short-axis. In this situation, the extent of AVPD is expected to be relatively low since it depends on the degree to which the epicardial short-axis changes during the cardiac cycle (indicated by transparent blue bands). Drawings are based on image data from [60] (**A**), [111,121] (**B**), and geometric calculations (**C**). Abbreviations: Ao = aorta; Epi-SAS = epicardial short-axis shortening; LAS = long-axis shortening; other abbreviations and colors as used before.

In the human heart, the reciprocating action of the atrial and ventricular chambers causes a reciprocating displacement of the AVP along the longitudinal axes of the ventricles during the cardiac cycle. During ventricular systole, the AVP becomes displaced toward the apex, while during ventricular diastole, the AVP moves back toward the cardiac base (Figure 14A). These movements of the AVP may be compared with the reciprocating movement of the piston unit in a piston pump [110]. It is regarded as the main mechanism for refilling the atrial and ventricular chambers in the human heart [60,104,105,106,107]. We should, therefore, put a final view on Kilner’s third postulated advantage, which says that a sigmoid routing of the cardiac flow path(s) may produce effects supporting the long-axis displacement of the AVP, while a linear routing of the cardiac flow path(s) may produce effects suppressing the long-axis displacement of the AVP, particularly in the exercising state.

Kilner’s concept is based only on reflections on the theoretical fluidic and dynamic consequences of a looped versus non-looped (linear) heart design. Although he noted that a two-chambered heart with a linear design is found in some invertebrates, such as the snail *Helix pomatia*, he did not consider observations on the pumping action of such hearts in his reflections on the postulated disadvantages of a linear heart design. This is astonishing since the published reports on the action of snail hearts consistently show that the pumping action of the two-chambered heart of *Helix pomatia* and other snails is characterized by an appreciable long-axis displacement of the AVP, which bears a striking resemblance to the reciprocating action of a traditional water lifting piston pump (Figure 14B) [111,112,113,114]. Moreover, it has been noted that this movement seems to play a prominent role in the refilling of the atrial and ventricular chambers of the snail heart [112,114]. At first sight, these observations seem to contradict Kilner’s idea that a linear arrangement of the heart chambers may hamper reciprocating long-axis displacement of the AVP during the cardiac cycle. One might argue, however, that Kilner and co-workers had speculated that this postulated disadvantage of a linear heart design might become effective, particularly in the exercising state, when an increase in cardiac output leads to a rise in ventricular recoil resulting from the ejection of blood into the great arteries. Snails are slowly moving animals that do not show a dynamically active behavior. The highest basal heart rates of *Helix pomatia*, or other land snails reported in the literature, were in the range of 50 to 60 beats per minute [112,115,116]. Most authors, however, reported lower heart rates, ranging from 20 to 45 beats per minute, even during activity or under stress conditions [113,117,118,119,120]. One, therefore, might speculate that the pumping performance of a snail heart is normally so low that, even in an exercising state, recoiling forces resulting from the ejection of blood (hemolymph) into the aorta cannot fully suppress the systolic long-axis movement of its AVP toward the aorta. Snail hearts, therefore, may not represent the best biological model for testing the validity of Kilner’s hypothesis. Despite this objection, however, I think that the observations on the pumping action of snail hearts demand a rethinking of the plausibility of Kilner’s theoretical reflections, which use the physics of a firing gun as a model for the postulated behavior of the ventricle of a linearly arranged two-chambered heart during the cardiac cycle. The questions are: (1), is a firing gun really a proper model for a ventricle that ejects blood into the aorta? (2), is it possible that Kilner and co-authors might have neglected one or more functionally important aspects when they made their theoretical reflections on the pumping action of a linearly arranged two-chambered heart? With regard to the first question, I should note that a gun chamber and its outlet (barrel) have rigid walls and, therefore, do not change their dimensions during shooting. Phenomena typical for contracting ventricles of vertebrate hearts, such as short-axis shortening and long-axis shortening, do not occur in this type of launching system. The design and behavior of a gun, therefore, bear little resemblance to that of the ventricle of a snail or vertebrate heart. The contracting ventricles of snail and vertebrate hearts rather show a strong resemblance to an inflated rubber balloon in which the air is rapidly squeezed out through the opening by the elastic contraction of the balloon. This physical model not only shows short-axis and long-axis shortening. It is, additionally, a simple and well-known teaching device to demonstrate Newton’s third law of motion. I, therefore, think that a balloon rocket is a much better model than a firing gun. With regard to the second question, I should note that Kilner and co-authors have made their theoretical reflections only for the action of hearts that were physically isolated from their normal environment (see Figure 3 in [24]). Such reflections neglect the fact that vertebrates, as well as snail hearts, are normally fixed at their venous and arterial poles to the wall of a pericardial cavity. The wall of the pericardial cavity, the blood vessels entering and exiting the heart, and the anatomical structures surrounding the pericardial cavity all together normally act as a frame for the fixation of the heart. This frame can absorb the recoil momentum resulting from the systolic ejection of blood so that the heart and its surrounding structures together may act as a non-recoil system. The behavior of such a non-recoil system can be demonstrated using the above-mentioned rubber balloon model. If the rubber balloon model is used without physical fixation, the inflated balloon contracts while it flies away in the direction opposite to the opening through which the air streams out of the balloon. If the rubber balloon model is used together with a frame to which the opening of the balloon is physically fixed, the elastic contraction of the balloon will squeeze out the air through the opening, but the balloon will remain at its place, where it undergoes short-axis and long-axis shortening.

The pumping action of snail hearts, as well as the behavior of the above-described rubber balloon model, strongly suggests that, even at strenuous exertion, a linear arrangement of the atrial and ventricular heart chambers may not hamper the long-axis displacement of the AVP. Thus, it appears that with regard to this functional feature, sigmoid routing of the cardiac flow path(s) may not provide a significant improvement compared to a linear heart design. Moreover, a geometrical analysis shows that, in hearts with an elongated ventricle, an appreciable displacement of the AVP during the cardiac cycle is expected to occur only in those hearts in which the AVP lies perpendicular or almost perpendicular to the ventricular long-axis (Figure 14A,B).

In vertebrates, such a situation is found either in hearts with a low degree of sigmoid routing of the flow path(s) (Figure 8A) or in hearts with high degrees of sigmoid routing of the flow path(s) (Figure 8C,D and Figure 9). The AVP of hearts with a moderate degree of sigmoid routing of the flow path lies perpendicular to the ventricular short axis (Figure 8B). In this situation, the extent of AVP displacement does not depend on the extent of long-axis shortening but depends on the degree to which the epicardial short-axis diameter changes during the cardiac cycle (Figure 14C). Compared to the length of the long axis of the ventricle(s), the epicardial short-axis diameter shows only small changes during the cardiac cycle [122]. Thus, AVP displacement will not significantly contribute to the pumping function of hearts with a ventricular L-turn loop, which drives the circulation of blood in many fish species, such as zebrafish.

Compared to hearts with a linear design or hearts with a high degree of sigmoid routing of the flow path(s), hearts with a ventricular L-turn loop show a further morphological feature that holds a potential disadvantage for their pumping function. The atrioventricular canal is arranged perpendicular to the outflow path of the ventricle so that, in cases of hearts with long atrioventricular valve leaflets, parts of the atrioventricular valve apparatus cross the ventricular outflow path (Figure 8B). Such a situation, for example, is found in the fish *Channa argus* and *Anabas testudines* (see Figure 1 in [123] and Figures 3 and 4 in [124]). This feature may represent a hemodynamically disadvantageous feature that may disturb the systolic bloodstream, thereby reducing the pumping efficiency of the heart. It may, additionally, predispose to the development of severe outflow tract obstructions as a consequence of pathologies of the atrioventricular valve. This situation seems to resemble that found in human heart pathologies with obstruction of the left ventricular outflow tract, in which a reduced angle between the mitral and aortic valves seems to play a prominent pathogenetic role [125]. Thus, a moderate degree of sigmoid routing of the cardiac flow path may not provide an advantage but rather provide potential disadvantages for the pumping function of mature vertebrate hearts. In view of this situation, it seems that sigmoid routing of the cardiac flow path(s) may not generally provide advantages for the pumping function of mature vertebrate hearts. It is a challenge for the future to clarify this situation by simulation studies that facilitate the comparison of the pumping performance between hearts with a linear design and those with various degrees of sigmoid routing of the cardiac flow path.

##### Implications for the Usage of Fishes in Cardiovascular Research

The above-described functional differences between fish hearts with a moderate degree of sigmoid routing and those with a high degree may have practical consequences for biomedical research since they suggest that fish hearts with a moderate degree of sigmoid routing of the flow path, such as the zebrafish heart, may not represent appropriate models for clarifying the genetic and molecular basis of the pumping function of the left ventricle of the human heart. It might be more suitable to use fish heart models with a high degree of sigmoid routing of the flow path, such as the heart of swamp eels (e.g., *Monopterus albus*) or the heart of gars (e.g., *Lepisosteus osseus*). The hearts of the latter two fishes not only show a U-turn loop of their ventricular flow path (Figure 8D and Figure 9) but additionally, are characterized by the presence of fibrous continuity between the atrioventricular and the arterial valve [56,58].

## 5. Summary and Conclusions

This article presents and discusses the currently available information on the looped design of vertebrate hearts and its implications for the cardiac pumping function. Thereby, emphasis is given to the following observations:

(1) the looped configuration of the flow path of vertebrate hearts is roughly characterized by an s-shaped (sigmoid) component and a bilaterally asymmetric (chiral) component.
(2) the fully looped heart tube of vertebrate embryos regularly shows sigmoid as well as chiral routing of its flow path.
(3) data from physical pump models suggest that the looped design of valveless embryonic heart tubes might improve the pumping efficiency.
(4) among the mature hearts of vertebrates, sigmoid and chiral routing of the flow path(s) is regularly found only in the multi-chambered hearts of lungfish and tetrapods. Here, the bilaterally asymmetric (chiral) anatomy is regarded as the main determinant of the alignment and functional or structural separation of the systemic and pulmonary flow paths.
(5) among the two-chambered hearts of fish, only sigmoid routing of the flow path is a regular feature, while a bilaterally asymmetric (chiral) configuration is **not** a regular feature. Among fish, we can find species with bilaterally asymmetric hearts as well as species with bilaterally symmetric hearts.
(6) the presence of bilateral symmetry in the mature hearts of some fishes seems to be the consequence of a process of back-rotation of the heart during the post-embryonic period of development.
(7) the presence of a nearly perfect bilateral symmetry in the high-performance hearts of several active fish, suggests that chiral routing of the cardiac flow path may not significantly improve the pumping function of two-chambered hearts. If the pumping function of two-chambered hearts may profit from a looped design, such a benefit should be attributed to the sigmoid routing of the cardiac flow path, which is a feature found in the mature heart of all vertebrates.
(8) the bilaterally asymmetric (chiral) anatomy of some fish hearts seems to represent no more than the solution to a packing problem.
(9) it was frequently stated that the evolution of the vertebrate heart was characterized by an increase in the degree of sigmoid routing of the cardiac flow path(s). The lowest degree was ascribed to the hearts of basal vertebrates (jawless fish). A moderate degree was ascribed to the hearts of jawed fish, while high degrees were ascribed exclusively to the hearts of lungfish and tetrapods. Original data from fish, however, show that, among jawed fish, we can find a spectrum of various degrees of sigmoid routing of the cardiac flow path. This spectrum ranges from low degrees, resulting from an atrial position that is dorso-caudal to the ventricle, to high degrees, resulting from an atrial position that is dorso-cranial to the ventricle. Thus, a high degree of sigmoid routing of the cardiac flow path(s) is not exclusively found in the mature hearts of tetrapods.
(10) it has been postulated that sigmoid routing of the cardiac flow path(s) may produce effects supporting the long-axis displacement of the AVP, while a linear routing of the cardiac flow path(s) may produce effects suppressing the long-axis displacement of the AVP, particularly in the exercising state. Data from snail hearts and physical models cast doubt on the validity of this hypothesis. The relation between the looped design and the pumping function of vertebrate hearts remains an enigma.

## Data Availability

No new data were created or analyzed in this study.
